# Characterization of the Core Temperature Response of Free‐Moving Rats to 1.95 GHz Electromagnetic Fields

**DOI:** 10.1002/bem.70013

**Published:** 2025-06-26

**Authors:** Nathan Bala, Rodney J. Croft, Robert L. McIntosh, Steve Iskra, John V. Frankland, Raymond J. McKenzie, Chao Deng

**Affiliations:** ^1^ University of Wollongong, School of Medical, Indigenous and Health Science, and Molecular Horizons Wollongong NSW Australia; ^2^ Wollongong Bioelectromagnetics Laboratory Wollongong NSW Australia; ^3^ University of Wollongong, School of Psychology Wollongong NSW Australia; ^4^ Australian Centre for Electromagnetics Bioeffects Research Wollongong NSW Australia; ^5^ Swinburne University of Technology, 6G Research and Innovation Lab Hawthorn VIC Australia; ^6^ Telstra Limited Melbourne VIC Australia; ^7^ Australian Mobile Telecommunications Association (AMTA) Canberra Australia

**Keywords:** body temperature, radiofrequency electromagnetic fields, radiotelemetry, rat

## Abstract

The present study investigated the core body temperature (CBT) response of free‐moving adult male and female Sprague Dawley rats, during and following a 3‐h exposure to 1.95 GHz radiofrequency electromagnetic fields (RF‐EMFs) within custom‐built reverberation chambers, using temperature capsules implanted within the intraperitoneal cavity and data transmitted via radiotelemetry. Comparing RF‐EMF exposures (at Whole‐Body Average‐Specific Absorption Rate [WBA‐SAR] levels of 0.1, 0.4, and 4 W/kg) to the sham exposed condition, we identified a statistically significant peak increase in CBT after 26 min of RF‐EMF exposure at 4 W/kg (+0.49°C), but not in the 0.1 or 0.4 W/kg conditions at the same timepoint. In the last 30 min of the RF‐EMF exposure, temperature was significantly increased in both the 4 W/kg (0.62°C) and 0.4 W/kg (0.14°C) conditions, but not 0.1 W/kg, when compared to sham. After 20 min following cessation of exposure, post temperature was still significantly higher in the 4 W/kg condition when compared to the sham (0.37°C), but not in either 0.1 or 0.4 W/kg. Based on our findings, it is apparent that rats can effectively compensate for increased thermal loads of up to 4 W/kg as the maximum temperature rise was substantially lower than 1°C. In addition, the elevated CBT during exposure in the 4 W/kg condition was significantly reduced immediately after exposure cessation, indicating that measures of CBT following RF‐EMF exposure cessation may not reflect maximum RF‐EMF‐mediated changes in the CBT of rats. Bioelectromagnetics. 00:00–00, 2025. © 2025 Bioelectromagnetics Society.

## Summary

- Exposure of 1.95 GHz electromagnetic fields at 4 W/kg WBA‐SAR increases core body temperature by 0.62°C.
- Rat thermoregulation can effectively compensate for thermal load due to exposure to RF‐EMF at 0.1 and 0.4 W/kg WBA‐SAR (maximum increase = 0.14°C).
- Post cessation measures of CBT in rats may not reflect maximum RF‐EMF‐mediated changes.

## 1 Introduction

Current evidence suggests that biological effects following exposure to mobile phone‐like frequencies of radiofrequency (RF) electromagnetic fields (EMFs) are primarily the result of a temperature rise in tissues (ICNIRP 2020). This has been investigated using animal models; however, the effects of RF‐EMF on rodent core body temperature (CBT) are still not sufficiently understood. The current practice for assessing the effect of RF‐EMF on rodent CBT is typically through the insertion of a rectal probe to provide a temperature reading immediately before or after exposure to RF‐EMF. For example, Kim et al.'s study measured CBT in anesthetized and non‐anesthetized rats after being exposed to 4 W/kg Whole‐Body Average‐Specific Absorption Rate (WBA‐SAR) for 8 h (Kim et al. 2020). During the exposure, elevations in CBT were reported when compared to a sham exposure, but the increase was still under 1°C in non‐anesthetized rats. However, this fails to monitor CBT changes during RF‐EMF exposure, and further, the procedure itself can cause stress to the rodents that can result in a rise of CBT that can take more than 60 min to return to baseline (Bae et al. 2007; Dallmann et al. 2006; Dangarembizi et al. 2017). Accordingly, it is likely that effects of RF‐EMF on CBT measurements following exposure have been influenced by the rectal thermometry procedure itself. There are less invasive options to measure rodent CBT, such as scanning a subcutaneous implanted microchip. However, such microchips are implanted just under the skin of the rodent, which does not provide a true indication of CBT. For example, microchip measurements can vary substantially with up to 3°C differences being reported due to factors other than CBT (Hankenson et al. 2018), such as air temperature and proximity of the microchip to brown adipose tissue (Crane et al. 2014).

To overcome such difficulties, wireless data loggers can be used; they can be implanted into the abdominal cavity of rodents to provide continuous real‐time measurements of rodent CBT itself (Cesarovic 2011; Kramer et al. 2001). Compared to rectal or subcutaneous methods, the use of wireless data loggers coupled with thermal probes in the animal core can provide insight into how RF‐EMF interacts with rodent CBT during the exposure period itself, and without any invasive procedures that are known to cause (thermal) artifacts. For example, a recent study implanted healthy Sprague Dawley rats with the iButton data logger, which then recorded rodent CBT during a 6‐h exposure to RF‐EMF in a reverberation chamber (RC) to see whether CBT changed over time (Kim et al. 2022). The baseline was determined by the first CBT measurement obtained and then compared to the averages of 10‐min intervals. During the 6‐h exposure period, they reported that 4 W/kg exposure did not cause any significant CBT changes compared to the sham. However, to accurately detect changes over time, a period of habituation is critical to allow rat CBT to return to a relative baseline after handling the rats. This is because handling the rat itself is known to cause stress, which can affect the CBT of rats (Dallmann et al. 2006). Without giving time for the rats to recover from stress‐related hyperthermia, both the sham and RF‐EMF groups would be affected by this and hence confound any RF‐EMF‐related CBT changes. Furthermore, Kim et al. (2022) performed rectal thermometry immediately after and before the exposure period, which is known to also cause stress to rats (Bae et al. 2007) and thus increase CBT (Dallmann et al. 2006).

Another study exposed male Sprague Dawley rats to RF‐EMF at either 4 or 0.4 W/kg, for both a single 6‐h period and for 3 consecutive days, using intraperitoneal CBT measurements (via iButtons) to investigate whether RF‐EMF affects CBT as a function of time (Ohtani et al. 2016). Contrary to Kim et al. (2022), they reported that RF‐EMF exposure at 4 W/kg caused a peak CBT increase of 1.1°C–1.4°C (compared to the sham), which occurred at approximately 10 min post RF‐EMF onset. The increase in CBT plateaued until the end of exposure before returning to baseline 30 min later. Even though they reported CBT measurements before and after the RF‐EMF exposure itself, the paper does not mention if the rats were in the cage before and after the exposure. This can confound results as a change in environment (such as transporting the rats to a different room) will cause them stress, which can last up to 1 h (Castelhano‐Carlos and Baumans 2009). Similarly, the health status of the rats may have confounded their results given that the first RF‐EMF exposure was performed 2 days after the surgical implantation of the iButton, which is less than the 1‐week recovery period that is recommended (Greene et al. 2007), and may explain why the animals were stated in the paper to have appeared sick. These factors raise the serious possibility that the reported increase in CBT was due to factors other than RF‐EMF exposure itself.

Although these studies are an improvement on the previous literature as they measured CBT changes over time using telemetric devices instead of rectal thermometry or subcutaneous microchips, further research is required to detect effects of RF‐EMF on rat CBT in the absence of factors such as handling and restraint. Our group has recently performed a study that obtained thermal measurements from free‐moving mice when exposed to RF‐EMF following the above conditions to address the concerns from previous studies (Sylvester et al. 2024). In that study, we reported that following exposure to 5 W/kg for 2 h, the maximum increase in mouse CBT was 0.4°C, which was far smaller than that due to animal handling and would not have been observable if confounding effects on CBT were not controlled for.

Another issue that has not been determined is the role of sex in RF‐EMF exposure changes to CBT. CBT is heavily dependent on an animal's surface area‐to‐mass ratio, as heat is more easily dissipated to the environment for animals with larger ratios. Larger animals have smaller ratios, and so, all else being equal, their CBT would be expected to increase more than that of smaller animals. It would thus be expected that males, with their smaller surface area‐to‐mass ratios, would increase CBT more than females when exposed to the same RF‐EMF power. This is indeed what was reported by Wyde et al. (2018) following exposure cessation using subcutaneous thermistors, but given the methodological issues with this approach (described above), it is important to verify these results.

As we currently do not know the extent to which CBT can change during an exposure to RF‐EMF, overall or as a function of sex, the present study assessed the CBT response of free‐moving male and female Sprague Dawley rats, before, during, and following a 3‐h exposure to 1.95 GHz RF‐EMF at various intensities; AniPill temperature capsules were used, implanted within the intraperitoneal cavity, and data transmitted post‐exposure via radiotelemetry. Since the RF‐EMF exposure guidelines designate 4 W/kg as the WBA‐SAR required to cause a 1°C increase in CBT, the present study included this WBA‐SAR value along with those that correspond approximately to the general public (0.08 W/kg) and occupational (0.4 W/kg) basic restrictions (ICNIRP 2020). Methodological limitations in previous studies have been addressed using telemetric capsules to provide real‐time CBT measurements, before, during, and after the RF‐EMF exposure, as well as the inclusion of a habituation period before rats are exposed to RF‐EMF. Additionally, rats are free‐roaming to simulate natural behavior; these features remove contamination from handling and restraint on CBT measures to provide a clear view of how the rat thermoregulatory system responds to RF‐EMF in custom‐built RCs.

## 2 Methods

### 2.1 Animals and Housing

Twelve male and 12 female Sprague Dawley rats (postnatal Day 23) were obtained from the Animal Resource Centre, Perth, Australia. Each rat was housed with another rat of the same sex in Tecniplast double‐decker rat individually ventilated cage housing units. The housing environment was maintained at a room temperature of 22°C ± 0.2°C and operated on a 12‐h dark/light cycle (0700–1900‐h light cycle, 1900–0700‐h dark cycle). Rats were allowed ad libitum access to water and standard laboratory chow. In the housing units, enrichment included tunnels, chew block, and nesting materials.

### 2.2 AniPill Implantation

The AniPill capsule (17.7 × 8.9 mm, 1.7 g, 1.12 cubic centimeter [implant volume]) was implanted into the intraperitoneal cavity of rats to provide measures of CBT. The capsule continuously recorded CBT throughout the course of the experiment, and data were downloaded post‐exposure using radiotelemetry. Rats were given a minimum of 13 days post‐surgery before the first exposure to RF‐EMF to avoid the impact of the surgery on the experiment (Greene et al. 2007; Schuler et al. 2009). The AniPill was shown to accurately report CBT changes in mice in another study that our lab performed (Sylvester et al. 2024).

### 2.3 Exposure System and Dosimetry

RF‐EMF exposure was provided by an RC, which allows for large‐scale, simultaneous exposure of multiple free‐roaming rats (confined only to nonmetallic cages). The RC delivered 1950‐MHz RF‐EMF exposure at four power levels, corresponding to WBA‐SAR levels of 0 (sham), 0.1, 0.4, and 4 W/kg. A full characterization of the dosimetry is provided previously (Iskra et al. 2025), which is described in brief below.

The RC was 1.45 m × 1.45 m × 1.45 m in internal chamber dimensions (manufactured by Compliance Engineering, Keysborough, Australia). Mode stirring was achieved using a combination of six antennas within the RC (placed centrally on each of the six sides) and three metallic stirrers (with blades of around 30‐cm length and 6‐cm width) placed in the corners of the floor of the RC. Statistical field uniformity in the central working volume (measuring 50 cm × 50 cm × 40 cm) of the RC was achieved by directing power to each antenna sequentially (10 s per antenna) with the stirrers rotating at approximately 30 revolutions per minute. The standard uncertainty in field strength uniformity in the unloaded chamber was determined through a set of spatial field strength measurements in the central working volume and found to be 0.92 dB. The RF‐EMF into the RC was provided by a signal generator, a digital step attenuator, an RF amplifier, and a six‐way coaxial switch feeding the signal into the antennas. Control software enabled the precise amount of power to be directed to each antenna to achieve a desired RF‐EMF exposure environment in the RC. Airflow for the rats consists of a mesh panel inserted into one of the sides of the RC for air input, another fan attached with a rubber mat base to a fine metallic mesh panel sits on the top of the RC for air output.

The relationship between the RF power delivered to the RC and the WBA‐SAR in exposed rats was determined through a calibration process involving the measurement of the RF‐EMF within the RC loaded with a predetermined number (*N*) of phantom rats each with the same mass (*m*) and through comparison with computational analysis of the exposure using the finite‐difference time‐domain (FDTD) technique and based on the same number and mass of models of phantoms in the RC and similarly arranged. Following the method described in Iskra et al. (2025), data obtained during calibration were used to generate a power function that linked the total mass *M* of phantom rats in the RC to the WBA‐SAR in the phantoms normalized to a 1‐W RF power (*P*_in_) delivered to the RC (Iskra et al. 2025). The total mass *M* takes account of the number of rats to be exposed and their growth with age. Calibration was performed under three conditions of *N* and *m*; *N* = 16 and *m* = 94.4 g (male or female rat at ~4 weeks of age), *N* = 8 and *m* = 245.4 g (male at ~6 weeks or female at ~9 weeks), and *N* = 4 and *m* = 449.9 g (male at ~9.5 weeks). The phrase “phantom rats” refers to plastic tubular bottles (with electrical conductivity assumed to be *σ* = 0 S/m, and relative permittivity *ε*_r_ = 3) containing tissue simulant liquid (with properties at 1950 MHz of *σ* = 1.4 S/m, *ε*_r_ = 40, similar to that used in Gong et al. [2017]), provided by EMC Technologies, Keilor Park, Australia (consisting of surfactant, water, and bactericide).

A power fit with the following equation was applied to the data obtained during calibration (with the coefficient of determination *R* ^*2*^ = 0.88):

(1) $$<\text{WBA}‐\text{SAR}>=0.289M^{-0.502}\times P_{\mathrm{in}}$$

Voxel models of the rats (obtained from Brooks Air Force Base, San Antonio, Texas, and consisting of 35 tissues) were used in computations to extrapolate the settings for the phantoms to rats. The desired WBA‐SAR (0.1, 0.4, or 4 W/kg) for a given *M* is obtained by simply scaling *P*_in_. From the data obtained during the calibration process, the SAR sensitivity, expressed as the WBA‐SAR normalized to 1 V/m incident *E*‐field strength ([W/kg]/[V/m]^2^), is 117 μW/kg/(V/m)^2^ for the 94.4‐g phantom, 78.2 μW/kg/(V/m)^2^ for the 245.4‐g phantom, and 62.8 μW/kg/(V/m)^2^ for the 449.9‐g phantom. The incident field strength required to produce a desired WBA‐SAR can be computed using the SAR sensitivity values. For example, an incident field strength of 226.2 V/m (rms) is required to produce a WBA‐SAR of 4 W/kg in a 245.4‐g rat.

The resultant Equation (1) can then be used by the experimenter, who, after measuring the total mass of (similarly aged) rats, can set the input power to the RC for a desired WBA‐SAR (0.1, 0.4, or 4 W/kg). Following the approach outlined in Iskra et al. (2025), the total expanded uncertainty (*k* = 2) in the estimation of WBA‐SAR, taking into account uncertainties related to measurement and computation of chamber performance, was found to be 3.86 dB, 95th percentile.

The AniPill used to provide continuous CBT measurements during the experiment may introduce potential uncertainty readings during the operation for the AniPill in the radio environment of the RC. Therefore, before the exposures started, the AniPill behavior was explored in pre‐experiments using rat phantoms and real rats to investigate potential interaction with the RF‐EMF under the proposed exposure conditions. During the pre‐experiments, the AniPill provided smoothly continuous temperature profiles during the RF‐EMF exposures without interactions between them observed. A similar setting in mice also showed no interactions between AniPill and RF‐EMF in the operating RC (Sylvester et al. 2024).

### 2.4 Experiment Design

**Figure 1:**
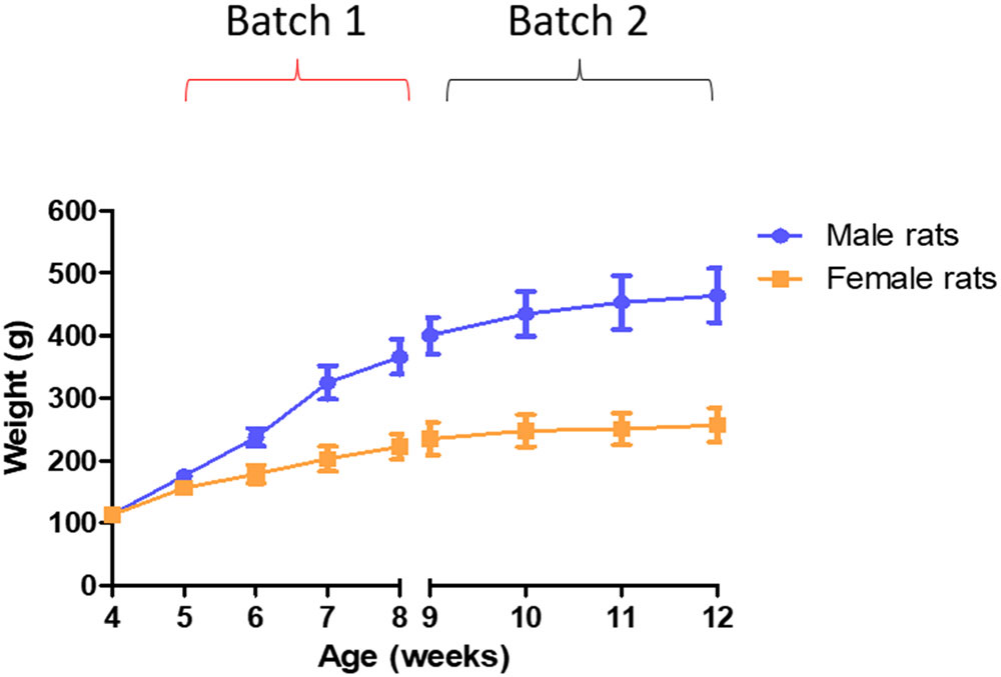
Bodyweight of rats of Batch 1 and Batch 2, data presented as mean ± SEM. There was a total of 24 rats used in this experiment. Batch 1 consisted of 8 male rats and 8 female rats, Batch 2 consisted of 4 male rats and 4 female rats for a total 12 male and 12 female rats.

The present study was not blinded. However, as temperature was recorded automatically (without any input from the experimenter), and as pilot testing demonstrated that experimenter effects on CBT had completely dissipated within an hour, a 1‐h habituation period was employed to ensure that blinding was not necessary. A total of four conditions were included in the present study: 0‐ (sham), 0.1, 0.4, and 4 W/kg WBA‐SAR. A total of 24 male (*n* = 11) and female (*n* = 12) rats were exposed once to each of these conditions for 3 h, on separate days. Originally, there were 12 males; however, the AniPill inside one rat stopped working after 1 week for unknown reasons. Therefore, this male rat was removed from the data analysis as it did not get enough temperature measurements. Male and female rats were exposed separately, such that during each exposure, there was only one sex of rats present in the chamber. Following cessation of exposure, rats were left in the chamber for a further 1‐h interval, making a total duration in the chamber of 5 h per exposure session. Rats completed each exposure condition (which was randomly allocated) throughout a 6‐day period; this was then repeated three times over a total period of 4 weeks to ensure that each rat was exposed to each condition four times. Rats were divided into two batches; 16 rats (8 female and 8 male) underwent exposures over the initial 4‐week period, whereas the remaining 8 rats (4 female and 4 male) underwent exposures after the first group was completed. The average weights of the rats in the two batches can be seen in Figure 1 and Table 1. The average weight of the rats in Batch 1 was 190.16 g ± 29.5 (mean ± SEM) for the female rats and 276.50 g ± 77.8 for the male rats. In Batch 2, the average weight of the female rats was 248.03 g ± 20.6 and 437.83 g ± 42.3 for male rats.

**Table 1:** Bodyweight of male and female rats in Batch 1 and Batch 2 (data were presented in grams ± SEM).

	Age (weeks)	Male rats (*n* = 12)	Female rats (*n* = 12)
	4	114.18 ± 4.26	113.17 ± 5.18
Batch 1	5	175.71 ± 10.76	156.20 ± 11.39
	6	237.80 ± 14.49	178.52 ± 14.81
	7	325.84 ± 27.13	203.25 ± 20.06
	8	366.66 ± 27.96	222.69 ± 20.57
Batch 2	9	400.28 ± 28.63	235.38 ± 25.94
	10	434.40 ± 35.69	248.41 ± 25.56
	11	452.69 ± 42.87	251.03 ± 25.81
	12	463.97 ± 43.89	257.32 ± 26.71

The protocol was as follows: Rats were transported from the holding room into the exposure room and put into exposure cages (14 × 31 × 17 cm; completely polysulfone one rat per cage, which allowed free movement and contained two paper towels to absorb urine with no food or water present). Exposure cages were then placed into the RC for the 1‐h habituation period (without RF‐EMF); this was immediately followed by the 3‐h RF‐EMF exposure and then 1‐h post‐exposure period. The environment did not change throughout the total 5 h in which rats were in the chamber (monitored by an AniPill placed inside the chamber). Throughout the 5‐h period, CBT was recorded once every 2 min, which was downloaded after the end of the post‐exposure period. In addition to the rats, a single AniPill was placed into a phantom that contained liquid that simulated the dielectric properties of biological tissue (weight specified as the average weight of male or female rats, separately), to record temperature changes coincident with the rat exposures but without the effect of thermoregulation.

### 2.5 Data Analysis

To determine if any time intervals were contaminated by nonbiological artifact, grand mean “temperature by time” profiles were created. This step was employed because it was noted that temperature changes over a 2‐min interval were sometimes larger than it would be deemed biologically plausible for CBT. Accordingly, if there was a change of −0.5°C or larger over a 2‐min interval, or if this change clearly contaminated the data over multiple points, then a linear interpolation was used to replace that/those values (using the preceding and subsequent biologically realistic timepoint).

**Figure 2:**
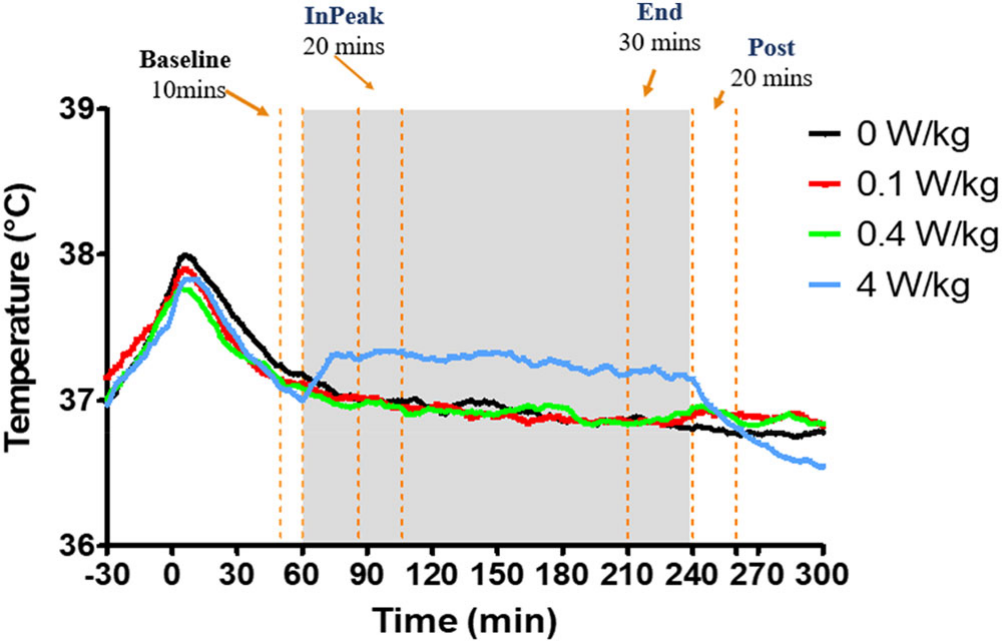
Data analysis of temperature measurements. This is the average of both male rats (*n* = 11) and female rats (*n* = 12) combined. Baseline was determined as the last 10 min before the start of the RF‐EMF exposure. “InPeak” was the average of a 20‐min period that started 26 min after RF‐EMF exposure begun. “End” was the average of the last 30 min before the end of the 3‐h RF‐EMF exposure. “Post” was the average of a 20‐min period after the end of the RF‐EMF exposure.

Due to the free‐moving nature of the rats (and thus substantial variability in CBT), we identified the most stable pre‐exposure interval to define as the baseline, and the interval that best represented the maximum temperature rise following onset of the RF‐EMF. For each of the male and female groups, the baseline was determined as the average 10‐min period immediately before the beginning of RF‐EMF exposure. The initial peak in temperature rise (“InPeak”) was the average of a 20‐min period that started 26 min after the beginning of the RF‐EMF exposure to provide an interval for the CBT of the rats to reach its maximum initial rise (CBT had plateaued at this time point; see Figure 2). The last 30‐min period just before the end of RF‐EMF exposure (“End”) was also averaged. For each rat and exposure condition, the baseline temperature was subtracted from each of “InPeak” and “End,” which provided the CBT change scores used in the statistical analyses (i.e., InPeak∆T and End∆T, respectively). To determine whether thermoregulatory processes were influencing the RF‐induced CBT changes, we computed the average of the first 20‐min following cessation of exposure (“Post”) and subtracted the End average from this to produce “Post∆T.”

### 2.6 Statistical Analysis

All statistical analyses were performed using SPSS (version 28, IBM, USA). All data were assessed for normality using a Shapiro–Wilk test. To determine whether RF‐EMF strength had an effect on rodent CBT, InPeak∆T, End∆T, and Post∆T data were analyzed separately using mixed design ANOVA with planned contrasts, comparing each of the (repeated measures) 0.1, 0.4, and 4 W/kg WBA‐SAR to the sham (0 W/kg). To determine whether sex affected any RF‐EMF‐induced changes on rat CBT, the interaction of each comparison with sex was assessed. The same analysis was run on phantom temperatures to determine the effect of RF‐EMF without the interaction of the rat thermoregulatory processes. Further, as the bodyweights of the rats first and second batches were different, which may influence the results, Batch (2 levels, 1;2) has been added as a factor to check whether these differences in body mass influenced the results.

### 2.7 Positive Control (Phantom)

To verify that the RF‐EMF exposure was successful, End∆T and Post∆T phantom temperature data were analyzed separately using mixed design ANOVA with planned contrasts, where repeated measures contrasts compared levels of Exposure (sham vs. each of 0.1, 0.4, and 4 W/kg WBA‐SARs).

## 3 Results

### 3.1 Positive Control (Phantom)

**Figure 3:**
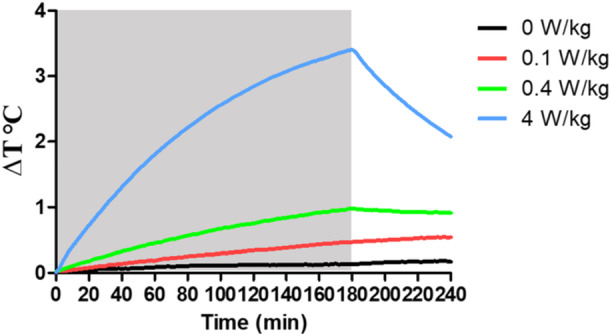
Change in temperature (relative to baseline) in rat phantoms (*n* = 2) during and following exposure cessation from 3‐h exposure to 1.95 GHz RF‐EMF at 0 W/kg (sham), 0.1, 0.4, and 4 W/kg WBA‐SAR. RF‐EMF exposure starts at 0 min; shaded area indicates RF‐EMF exposure is ON. Relative to the sham, each of the RF‐EMF exposures resulted in significant temperature rises at last 30‐min interval of the exposure; 4 W/kg was the only strength to cause a significant decrease at the 30‐min interval immediately after exposure cessation.

As can be seen in Figure 3, phantom temperature did not change appreciably over time in the sham condition, whereas it continually increased over the course of the 3‐h exposure interval as a function of exposure magnitude, in which the maximum temperature (EndΔT) for each condition was 0.44°C (0.1 W/kg) (*F*[1, 11] = 6.125, *p* = 0.031), 0.93°C (0.4 W/kg) (*F*[1, 11] = 28.034, *p* < 0.001), and 3.28°C (4 W/kg) (*F*[1, 11] = 562.19, *p* < 0.001). After RF‐EMF ceased, temperature decreased rapidly in the 4 W/kg condition (*F*[1, 11] = 40.44, *p* < 0.001), while relatively smaller reductions were observed in 0.4 W/kg (*F*[1, 11] = 1.483, *p* = 0.249), and there was a slight increase in temperature in 0.1 W/kg (*F*[1, 21] = 0.1.83, *p* = 0.203) (Figure 3). All values for EndΔT and PostΔT are given in Table 2.

**Table 2:** InPeakΔT, EndΔT, and PostΔT values (°C) at a WBA‐SAR of sham, 0.1, 0.4, and 4 W/kg for phantom (*n* = 2), male rats (*n* = 11), female rats (*n* = 12), and the average of male and female data (*n* = 23) when compared to its baseline (/B). Data are presented as mean (standard error of the mean). M + F refers to averaged male and female data.

		0 W/kg (sham)	0.1 W/kg	0.4 W/kg	4 W/kg
InPeakΔT/B	M + F	−0.20 (0.04)	−0.18 (0.05)	−0.23 (0.05)	0.29 (0.04)
	Male	−0.20 (0.07)	−0.14 (0.06)	−0.12 (0.05)	0.26 (0.05)
	Female	−0.21 (0.06)	−0.22 (0.08)	−0.33 (0.08)	0.31 (0.06)
EndΔT/B	Phantom	0.13	0.44	0.93	3.29
	M + F	−0.46 (0.06)	−0.40 (0.06)	−0.32 (0.07)	0.16 (0.06)
	Male	−0.35 (0.08)	−0.27 (0.10)	−0.22 (0.09)	0.12 (0.08)
	Female	−0.56 (0.08)	−0.52 (0.07)	−0.42 (0.09)	0.20 (0.08)
PostΔT/B	Phantom	0.18	0.04	0.03	−0.16
	M + F	−0.02 (0.03)	0.06 (0.03)	−0.04 (0.03)	−0.27 (0.03)
	Male	−0.05 (0.03)	0.06 (0.04)	0.05 (0.02)	−0.23 (0.05)
	Female	0.02 (0.05)	0.05 (0.04)	−0.12 (0.04)	−0.30 (0.02)

### 3.2 Rat CBT

#### 3.2.1 InPeakΔT

**Figure 4:**
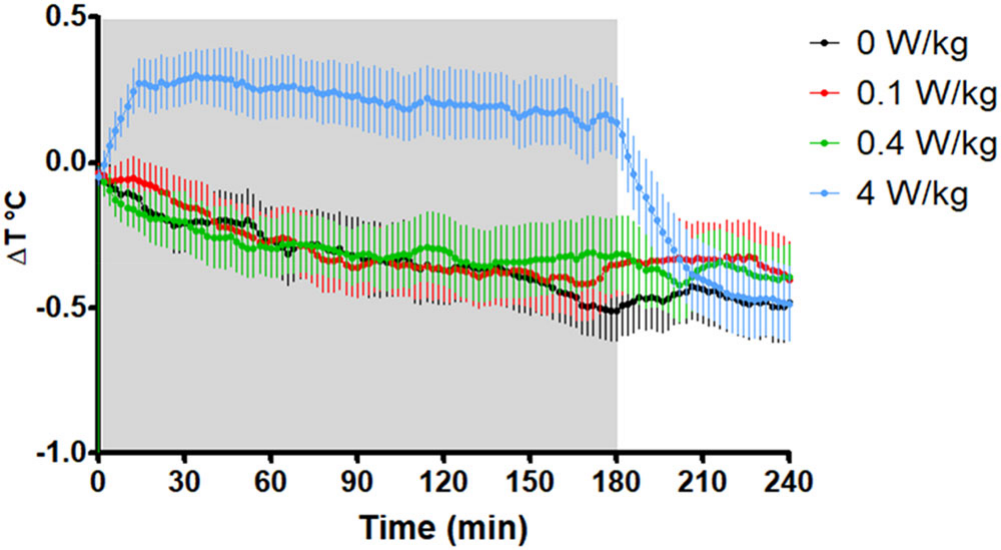
Change in temperature from baseline in rats (11 males and 12 females averaged) during 3‐h exposure to 1.95 GHz RF‐EMF at 0 W/kg (sham), 0.1, 0.4, and 4 W/kg WBA‐SAR. Shaded areas indicated RF‐EMF exposure is ON; error bars represent ± standard error of the mean. Relative to the sham, only 4 W/kg significantly increased CBT after a 26‐min interval of exposure, both 0.4 and 4 W/kg caused a significant increase within the last 30‐min interval. In the 20 min following post cessation, only 4 W/kg significantly increased CBT when compared to sham.

Compared to sham, rat CBT did not differ in the 0.1 W/kg (*F*[1, 19] = 0.277, *p* = 0.604) or 0.4 W/kg (*F*[1, 19] = 0.118, *p* = 0.735) conditions, whereas it was larger in the 4 W/kg condition (*F*[1, 19] = 99.59, *p* < 0.001) (Figure 4, Tables 2 and 3). No interaction between sex and any of these contrasts was found (*F*[1, 19] ≤ 3.46, *p* > 0.077), and there was no effect of sex (*F*[1, 19] = 1.13, *p* = 0.252) or batch (*F*[1, 21] = 0.22, *p* = 0.884).

**Table 3:** InPeakΔT, EndΔT, and PostΔT values (°C) at a WBA‐SAR of 0.1, 0.4, and 4 W/kg relative to sham (/S represents compared to sham). Data are presented as mean (standard error of the mean). M + F refers to averaged male (*n* = 11) and female (*n* = 12) data.

		0.1 W/kg	0.4 W/kg	4 W/kg
InPeakΔT/S	M + F	0.03 (0.06)	−0.02 (0.06)	0.49 (0.05)
	Male	0.07 (0.10)	0.09 (0.08)	0.46 (0.08)
	Female	−0.01 (0.08)	−0.13 (0.08)	0.52 (0.06)
EndΔT/S	M + F	0.06 (0.06)	0.14 (0.06)	0.62 (0.07)
	Male	0.08 (0.08)	0.13 (0.09)	0.47 (0.10)
	Female	0.04 (0.09)	0.15 (0.08)	0.76 (0.09)
PostΔT/S	M + F	0.07 (0.04)	−0.02 (0.04)	−0.25 (0.04)
	Male	0.11 (0.05)	0.10 (0.03)	−0.18 (0.05)
	Female	0.04 (0.05)	−0.13 (0.06)	−0.31 (0.05)

#### 3.2.2 EndΔT

Compared to sham, rat CBT did not differ in the 0.1 W/kg (*F*[1, 19] = 2.018, *p* = 0.172) condition (Figure 4, Tables 2 and 3). However, changes in rat CBT were larger in the 0.4 W/kg (*F*[1, 19] = 5.224, *p* = 0.034) and 4 W/kg (*F*[1, 19] = 67.781, *p* < 0.001) conditions. However, it should be noted that there was a trend level interaction between sex and “4 W/kg versus sham” comparison as the effect was larger in females (*F*[1, 19] = 3.78, *p* = 0.067). Additionally, there was also an interaction between Sex, Batch, and Strength in the “0.4 W/kg versus sham” comparison (*F*[1, 21] = 6.456, *p* = 0.020).

#### 3.2.3 PostΔT

Compared to sham, rat CBT differed in both the 0.1 W/kg (*F* = [1, 19] = 6.844, *p* = 0.17) and in the 4 W/kg condition (*F* = [1, 19] = 36.071, *p* < 0.001), in the 0.4 W/kg condition, female rat CBT displayed a larger reduction compared to the male rats (*F* = [1, 19] = 7.474, *p* = 0.009) (Figure 4, Tables 2 and 3). No effect of Batch was found (*F* = [1, 19] = 0.719, *p* = 0.407).

## 4 Discussion

The aim of this study was to characterize the CBT of free‐moving rats (M + F) when exposed to RF‐EMF at 0.1, 0.4, and 4 W/kg WBA‐SAR, compared to a sham exposure. We found statistically significant increases in rat CBT following exposure to 4 W/kg (+0.62°C) and 0.4 W/kg (+0.14°C). The initial temperature rise in the 4 W/kg (+0.49°C) condition was relatively maintained until the end of RF‐EMF exposure period (+0.62°C). We did find a trend‐level interaction during this period showing that the temperature increase compared to sham was larger in the female rats than in the male rats. There was also a small but significant increase in the 0.4 W/kg condition at the end of exposure (+0.14°C). Following exposure cessation, there were differences in rat CBT in both the 0.1 and 4 W/kg conditions for both males and female rats compared to sham. In the 0.4 W/kg condition, post cessation only female rats displayed a difference in rat CBT when compared to sham. No other statistically significant differences were observed. It is worth to note that our results only pertain to 5–12‐week‐old rats at this particular bodyweight range (Figure 1, Table 1), different species of rodents along with rats with different bodyweights may produce different results from those reported in this study.

Even though we found a robust elevation in CBT following 4 W/kg WBA RF‐EMF exposure, the average maximum temperature increase was only 0.62°C (relative to sham). It is thus evident that 4 W/kg was not a significant thermal stressor in this group of rats. There are several possible reasons for this. The thermoregulatory system of the rat may have been able to compensate for the extra energy absorbed by exposure to RF‐EMF, given that the temperature increases in the phantoms (which weigh the same as the rats but do not have active thermoregulatory processes) were an order of magnitude larger (+3.28°C). Following cessation of exposure, CBT of the phantoms dropped immediately afterwards by 0.16° (still 3.12°C above baseline) as there was no longer any RF‐EMF energy to heat up the tissue. Rat CBT also dropped following cessation (−0.25°C, relative to sham); during the initial 10 min following exposure cessation rat CBT dropped by 0.016°C/min, and then by 0.011°C/min in the following 10 min; this temperature decline can be seen in Figure 4. The thermoregulatory system returned rat CBT to levels similar to sham 1 h after exposure cessation. That is, rats' thermoregulatory system employs mechanisms such as vasodilation of the tail‐skin and skin of the feet, evaporation by spreading saliva, and voluntary urination to help reduce RF‐EMF elevation in CBT (Wanner et al. 2015). A study from our laboratory reported that C57BL/6 mice exposed to RF‐EMF at 5 W/kg using similar methodology resulted in a peak (0.37°C increase) in CBT after 16 min of exposure, which then largely dissipated over the remainder of the exposure period (Sylvester et al. 2024). When we consider that the rats in this study displayed their maximum increase in CBT towards the end of the exposure, it is clear that CBT continues to be elevated for longer in rats, likely due to their smaller surface area‐to‐mass ratio.

Pilot studies for the NTP, exposed rats to multiple levels of 900 MHz RF‐EMF within an RC, for approximately 9 h a day (daily exposures over a period of 18 h and 20 min with continuous cycling of 10 min on and 10 min off, leading to actual exposure for 9 h and 10 min) for 5 days (Wyde et al. 2018). They reported that exposure to 4 W/kg did not cause excessive increases to CBT in young and aged female/male rats, and pregnant rats, which they defined as a CBT rise of > 1°C; however, these thermal measurements were taken from interscapular, subcutaneously‐implanted microchips 1–2 min after the end of exposure (during the 10‐min off exposure period), which do not measure CBT unless thermal equilibrium has been reached (which there was not sufficient time to occur). To address this issue, the present study used the AniPill to obtain CBT measurements in rats during the RF‐EMF exposure period. This measurement approach also revealed that following cessation of RF‐EMF exposure, the rat's CBT dropped by 0.016°C/min in the first 10 min. This finding is important because the NTP report states that their temperature readings were taken 1–2 min after exposure cessation (Wyde et al. 2018), which, if their rats exhibited a similar decline in temperature, would mean that they would have underestimated CBT by an average of 0.016°C to 0.032°C. Further, as they measured subcutaneous and not CBT, their underestimation would likely have been even larger.

There are two studies that have exposed rats to RF‐EMF at 4 W/kg inside an RC while recording CBT using a similar device to the AniPill. The first study investigated rodent CBT in response to 1760 MHz RF‐EMF exposure at 4 W/kg for 6 h (Kim et al. 2022). This study used the iButton (telemetric device) to record CBT changes over time during the RF‐EMF exposure. They reported no significant changes to CBT from the 4 W/kg condition when compared to the sham group. However, they did not control for baseline variability by using an extended acclimatization period. Given that the transporting‐ and handling‐induced temperature rise of rat CBT, which was twice as large as the CBT changes from the 4 W/kg exposure (see Figure 2, a significant CBT increase at 0 min time point), and that such effects will not be consistent across testing sessions, it is important to control for the potential impact of the transporting/handling rats on CBT in RF‐EMF studies. This study employed a 1‐h habituation period to minimize the transporting/handling effects on rat CBT. With this in mind, we would expect any increase in rat CBT from handling to dissipate after several hours to which is not shown; therefore, we can only speculate that a difference in exposure environment may be the reason for no significant difference between the 4 W/kg and sham group. The bodyweight of the male rats in Kim et al.'s study was approximately 339 g (Kim et al. 2022). This may be another reason why our results differ since the present study's male rats were approximately 276 g in Batch 1 and 437 g in Batch 2.

The second study by Ohtani et al. exposed eight 6‐week‐old male Sprague Dawley rats to 0.4 or 4 W/kg 2.14 GHz RF‐EMF for 1 or 3 days (Ohtani et al. 2016). The study also utilized the iButton to record CBT changes over time, where CBT was reported to increase up to 1.5°C during the exposure to 4 W/kg and plateaued until the cessation of RF‐EMF. The present study also reported increases in rodent CBT following 4 W/kg but not of the same magnitude. The larger increase in rat CBT that they reported could be influenced by several factors. The first is that the surgical implantation of the wireless data logger was performed 2 days before the first exposure to RF‐EMF. Rats were reported to be sick following the surgery, which was then reported to subside after 3 days. However, the after‐effects from surgical procedures such as this can cause physiological changes for up to several days, which may affect the thermoregulation system (Greene et al. 2007). The present study allowed a recovery time of 13 days following the surgery to avoid any aftereffects of the procedure itself affecting rat CBT. Additionally, 6‐week‐old male rats were used in Ohtani et al. (2016); possibly, their rats had a similar bodyweight to Batch 1 male rats in our study. Therefore, the bodyweight is most possibly not a reason to explain the different results between Ohtani et al. (2016) and this study. Lastly, the exposure time in Ohtani et al. (2016) was longer than the present study (6 h vs. 3 h). Since the present study does not go beyond 3 h, it is hard to say what could happen if the RF‐EMF exposure continued.

The complexity underlying changes to physiological processes following RF‐EMF exposure is still only partially understood, and further investigation is required. It is interesting that, once RF exposure ceased, the increased rat CBT dropped immediately, likely due to the lack of incoming RF energy, which is consistent with Ohtani's study (Ohtani et al. 2016). Unexpectedly, there was a trend level interaction in the EndΔT/S period with a larger effect of RF on female rat CBT, which appears to be inconsistent with the known relationship between the surface area‐to‐mass ratio and temperature rise from RF‐EMF. That is, we would expect that male rats (with a small surface area‐to‐mass ratio) would produce a larger temperature rise. One possible explanation is that for rats used for research under standard laboratory conditions (temperature around 20°C–25°C), there are sex differences in thermoregulation such as increased UCP1 levels in Brown Adipose tissue in female rats. Since we know that UCP‐1 is involved in heat production, it would be interesting to see whether this has been upregulated in rats exposed to the higher levels of RF‐EMF (Fernández‐Peña et al. 2023). Therefore, further studies would be valuable to investigate the effects of RF‐EMF on the thermoregulatory system in rats.

Regardless, future studies that investigate the CBT response of free‐moving rats to RF‐EMF exposure need to include a habituation period before the onset of RF‐EMF exposure. This is due to the initial temperature variation from handling being far larger (~1°C) than the maximum temperature change that was induced by RF‐EMF (up to 4 W/kg), and which takes up to an hour to return to a relative baseline. This is to ensure that any stressful procedures such as handling will not influence CBT changes while RF‐EMF is being delivered to the rats. We demonstrate that the inclusion of habituation/post cessation period combined with the AniPill is an improvement on previous methodology to investigate CBT changes in rats following RF‐EMF exposure.

## 5 Conclusions

In this group of rats, moving freely within cages, RF‐EMF exposure at 4 W/kg WBA‐SAR induced an average maximum CBT increase of 0.62°C in male and female rats relative to the sham exposure. The initial increase in CBT occurred during the 26 min following the start of the RF‐EMF exposure, before reaching a plateau that lasted until cessation of exposure. The rat CBT dropped immediately once RF‐EMF had ceased (−0.25°C after cessation of 4 W/kg). When exposed to 0.4 W/kg, the maximum temperature increase in this group of rats was 0.14°C, while there was no change observed from exposures to 0.1 W/kg. We also saw that in some RF‐EMF conditions, exposures had a larger impact on female rat CBT towards the end of the exposure period and immediately following cessation. Once RF‐EMF exposure has ceased, it should be noted that CBT measurements post‐exposure do not reflect the increase in rat CBT induced during the 3‐h exposure period to RF‐EMF. Our findings suggest that rats can effectively compensate for the increased thermal loads at exposures up to and including 4 W/kg, as the maximum temperature increase was substantially lower than that found in the phantoms, as well as being substantially lower than the putative 1°C adverse health effect threshold employed by ICNIRP (ICNIRP 2020). Future studies need to have a habituation period before investigating the thermal response to RF‐EMF as the impact of handling was larger than the highest SAR the rats were exposed to.

## Conflicts of Interest

Robert L. McIntosh is a current employee of a telecommunications company; Raymond J. McKenzie is a consultant to the Australian Mobile Telecommunications Association; and Steve Iskra, Raymond J. McKenzie, and John V. Frankland are former employees of a telecommunications company. All authors read and approved the paper for publication.
